# NLRP3 inflammasome-dependent pyroptosis is proposed to be involved in the mechanism of age-dependent isoflurane-induced cognitive impairment

**DOI:** 10.1186/s12974-018-1299-x

**Published:** 2018-09-14

**Authors:** Lei Yin, Fangping Bao, Jing Wu, Kuanyu Li

**Affiliations:** 1 Department of Anesthesiology, Shanghai Pudong New Area People’s Hospital, Shanghai, 201200 China; 2 Wannan Medical College, Anhui, 241003 China; 3 Department of Anesthesiology, The First Affiliated Hospital, College of Medicine, Zhejiang University, Hangzhou, 310003 China; 4 Jiangsu Key Laboratory of Molecular Medicine, Medical School of Nanjing University, Nanjing, 2100093 China

**Keywords:** Isoflurane, Cognition, NLRP3, MCC950, Pyroptosis

## Abstract

Wang Z et al. recently published a paper, titled “Critical role of NLRP3-caspase-1 pathway in age-dependent isoflurane-induced microglial inflammatory response and cognitive impairment”. The finding in this paper is consistent with our previous study on NLRP3-caspase-1 pathway. Here, we propose that NLRP3 inflammasome-dependent pyroptosis may be involved in the mechanism of age-dependent isoflurane-induced cognitive impairment and discuss that inhibiting NLRP3 inflammasome activation with a novel inhibitor MCC950 may ameliorate age-dependent isoflurane-induced neuro-inflammation.

Letter to the editor

Recently, we read with great interest the article titled “Critical role of NLRP3-caspase-1 pathway in age-dependent isoflurane-induced microglial inflammatory response and cognitive impairment” by Dr. Wang Z et al. [1], who concluded that NLRP3 priming status in aged mouse brain may be involved in isoflurane-induced hippocampal inflammation and cognitive impairment. We appreciate this study and would like to present our opinion on it.

The NLRP3 inflammasome-caspase-1 pathway has been implicated in several metabolic and inflammatory diseases [2, 3]. It is also proposed to be a possible pathogenic mechanism for general anesthesia-induced neuro-inflammation and cognitive impairment [4, 5]. We have previously shown that isoflurane induces activation of NLRP3, cleavage of caspase 1, and increase of IL-1β and TNF-α levels in the hippocampus of aging mice and supposed that inhibiting the NLRP3 inflammasome might have therapeutic merit for ameliorating general anesthesia-induced cognitive deficits [5] as cited in Wang’s study. However, the selective inhibitor of NLRP3 inflammasome was lacking. Interestingly, in Wang’s study, they demonstrated that treatment of Ac-YVAD-cmk, an inhibitor of NLRP3-caspase-1, reversed isoflurane-induced microglial inflammatory response and cognitive impairment in aged mice [1]. This finding is a very important evidence for supporting that NLRP3-caspase-1 pathway is involved in the mechanism of general anesthesia-induced cognitive impairment. However, Ac-YVAD-cmk is a specific inhibitor of caspase-1, not of NLRP3 inflammasome. It is uncertain whether cleavage of caspase-1 directly results from NLRP3 inflammasome activation and results in the secretion of the pro-inflammatory IL-1β and IL-18 in Wang’s study [1, 6, 7]. The treatment of aging mice with NLRP3 inflammasome inhibitor will be needed. MCC950 is a highly potent specific NLRP3 inhibitor and was first introduced as a specific anti-inflammatory compound since 2015 [8]. The novel compound MCC950 has been clearly shown to be neuroprotective in multiple neurological disorders, including ischemic and degenerative diseases [9, 10]. Although MCC950 has not been used in general anesthesia issues, we think that it is promising as a therapeutic compound for treatment of general anesthesia-induced neuro-inflammation and cognitive impairment.

Pyroptosis is a novel inflammatory form of programmed cell death that has been discovered and verified recently. This type of cell death is characterized to be NLRP3 inflammoasome-caspase-1 dependent [11–13]. Recent advances strongly hint that pyroptosis plays a regulatory role in many infectious and noninfectious diseases [11], and MCC950 can inhibit NLRP3 inflammasome to further prevent pyroptosis [12]. However, little is known about the functions of pyroptosis in general anesthesia-induced neurotoxicity, and thus, an effective intervention, MCC950, is emerging. Wang’s study found that isoflurane activated NLRP3-caspase-1 pathway and increased the secretion of IL-18 and IL-1β in cells pretreated with lipopolysaccharide and suggested that isoflurane induces age-related hippocampal inflammation through NLRP3-caspase-1 pathway. In this regard, we present a hypothesis that pyroptosis plays a critical role in the mechanism of isoflurane-induced cognitive impairment and MCC950 inhibits the NLRP3 inflammasome-dependent pyroptosis in isoflurane anesthesia to improve the cognition.

Collectively, we present our hypothesis that NLRP3 inflammasome-dependent pyroptosis may be involved in the mechanism of age-dependent isoflurane-induced cognitive impairment, and inhibition of the NLRP3 inflammasome activation with MCC950 may ameliorate age-dependent isoflurane-induced neuro-inflammation. Future studies are needed to confirm the hypothesis.

We again compliment the authors on their excellent article and thank them for their contribution to it.

## Abbreviations

IL-18: Interleukin-18
IL-1β: Interleukin-1β
NLRP3: NOD-like receptor protein 3
TNF-α: Tumor necrosis factor-α
